# Detection and Compensation of Degeneracy Cases for IMU-Kinect Integrated Continuous SLAM with Plane Features †

**DOI:** 10.3390/s18040935

**Published:** 2018-03-22

**Authors:** HyunGi Cho, Suyong Yeon, Hyunga Choi, Nakju Doh

**Affiliations:** 1 School of Electrical Engineering, Korea University, Seoul 02841, Korea; hgcho91@gmail.com (H.C.); dkhyung@gmail.com (H.C.); 2 Naver Labs, Gyeonggi-do 13494, Korea; suyong.yeon@naverlabs.com; 3 TeeVR Inc., Seoul 02857, Korea

**Keywords:** degeneracy, inertial measurement unit (IMU)-Kinect integration, plane feature, pose estimation, simultaneous localization and mapping (SLAM)

## Abstract

In a group of general geometric primitives, plane-based features are widely used for indoor localization because of their robustness against noises. However, a lack of linearly independent planes may lead to a non-trivial estimation. This in return can cause a degenerate state from which all states cannot be estimated. To solve this problem, this paper first proposed a degeneracy detection method. A compensation method that could fix orientations by projecting an inertial measurement unit’s (IMU) information was then explained. Experiments were conducted using an IMU-Kinect v2 integrated sensor system prone to fall into degenerate cases owing to its narrow field-of-view. Results showed that the proposed framework could enhance map accuracy by successful detection and compensation of degenerated orientations.

## 1. Introduction

Plane features have been widely used for simultaneous localization and mapping (SLAM) of indoor environments due to the following two reasons: (1) there are abundant planes in man-made indoor spaces; and (2) sensor noise can be sufficiently reduced by conventional plane extraction algorithms.

However, the use of plane features can fall into degenerate cases when the number of linearly independent information of detected planes is insufficient for pose estimation. In other words, if less than three independent planes are detected from two consecutive 3D poses, the relative geometric relationship cannot be fully estimated. For example, for a long and flat corridor, the amount of translation along the corridor’s direction cannot be detected even with wide field-of-view (FoV) LiDAR sensors. If one uses narrow FoV sensors such as Kinect (Microsoft), these degeneracies are frequently encountered, even in sufficiently complex indoor spaces [1].

In this paper, we proposed a way to handle these degeneracy cases using a commercially efficient sensor combination: a Kinect v2 with an inertial measurement unit (IMU). As is well known, a lot of research has been conducted regarding the degeneracy problems as in [2,3,4,5,6,7,8,9,10,11,12,13,14,15,16,17,18,19,20,21]. This research can be categorized in two ways: (1) directly eliminating degeneracy by employing constrained motion [2,3], sensor fusion [4,5,6,7], additional or new types of feature(s) [8,9,10,11,12,13], online calibration [14], determining system configurations [15], or probabilistic depth map [16]; and (2) indirectly reducing the effect of degeneracy by minimizing null-space components in the direction of the degeneracy. Pathak et al. [17] have proposed a method that can substitute null-space components by odometry reading. Our method is complementary to the work of Pathak et al. in that our method considers the orientation while Pathak et al. handle the translation. Recently, Zhang et al. [18] have suggested a method that covers degeneracies both in rotation and translation. However, their method is designed for LiDAR, which has a wider FoV than Kinect v2 used in the present study.

The contribution of this paper consists of two parts. One is detection of degeneracy between two sets of plane features by evaluating ratios of eigenvalues of a second moment matrix. Here, we used the fact that degeneracy could induce significant uncertainty. This uncertainty is reflected in the second moment matrix as a relatively small or infinitesimal eigenvalue. The other is to compensate orientations that encountered degeneracy by projecting the orientation value of IMU to those of corresponding plane features. This is possible due to orthogonal properties of the second moment matrix’s subspaces. This enables projection only to the degenerate direction.

**Figure 1:**
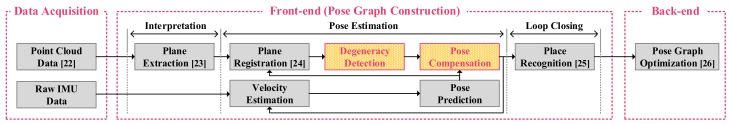
Schematic diagram of our proposed continuous 3D SLAM framework [22,23,24,25,26]. Yellow-colored boxes are contributions of this paper.

On the basis of the proposed detection and compensation method, we implemented a seamless 3D SLAM as shown in Figure 1 using a Kinect v2 and IMU. Experiments were conducted in a typical indoor place where more than 30% of place indices corresponded to the degeneracy case. Results showed that the proposed framework enhanced map accuracy compared to the method of Pathak et al. [17] by successfully detecting and compensating degenerated orientations.

This paper is organized as follows. Details of the algorithm pipeline (as shown in Figure 1), including two of our contributions, are explained in Section 2. Experimental results and their analyses are given in Section 3. Conclusions are provided in Section 4.

## 2. Seamless 3D SLAM Framework

The proposed pipeline in Figure 1 has the conventional Graph SLAM structure with three main modules: data acquisition, front-end construction, and back-end optimization. In the first block (data acquisition), raw 3D point cloud data from Kinect v2 and 6-DoF (degrees of freedom) measurements from the IMU are acquired. Those data are passed to the second block and plane features are extracted by using algorithms in [23,24]. Here, an i-th plane feature ($\Pi_{i}$) consists of unit normal ($n_{i}\in R^{3\times 1}$), which is orthogonal to the surface, and a perpendicular distance ($d_{i}$) from the origin to the surface.

At this point, the degeneracy can be induced if less than three independent plane-pairs are used. As these degeneracies provide clear evidence in the second moment matrix, the degeneracy detection algorithm (in Section 2.1, our first contribution) can analyze the matrix and pinpoint the degeneracy direction. Then, the pose compensation algorithm (in Section 2.2, our second contribution) can compensate for the degeneracy by projecting it to that of the IMU. Remaining procedures are conventional loop detection and back-end optimization adopted from methods in [25,26], respectively.

### 2.1. Degeneracy Detection Algorithm

**Figure 2:**
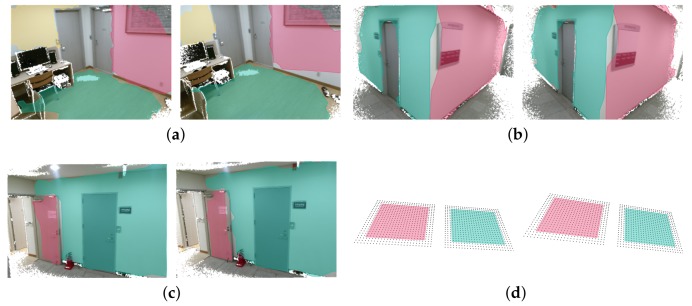
*l̃eft* to *r̃ight* point clouds for possible situations of plane feature based pose estimation. (**a**) 3-pair correspondence; (**b**) 2-pair correspondence; (**c**) fake 2-pair correspondence; (**d**) 1-pair correspondence with identical normals.

Relative pose estimation with plane features are illustrated in Figure 2, where sets of plane features are associated with each other. When there are more than three pairs of planes (as in Figure 2a), all 6-DoF parameters (3-DoF for rotation and 3-DoF for translation) can be estimated. However, when one pair of planes with identical normals exist (as in Figure 2d), 1- and 2-DoF degeneracy arises for rotation and translation, respectively. These two cases are out of the scope of this paper as noise (from motion as well as sensors) either does not significantly affect pose estimation or does not exist. However, in the case of 2-pair correspondence, noises can convert a 1-pair correspondence into a false 2-pair correspondence as shown in Figure 2c. The role of this subsection is to propose an algorithm that can discriminate false 2-pair correspondence from real 2-pair correspondence cases.

For that purpose, we adopted the second moment matrix, in which the rank and ratio of eigenvalues indicate the number of linearly independent plane correspondences and distinctiveness among independent correspondences, respectively.

Let us assume that there are corresponding planes ($\Pi_{1:N}$) between two consecutive frames from *l̃eft* to *r̃ight*. On the *l̃eft* coordinate, a stack of unit normals (${}^{l}n_{1:N}$) of $\Pi_{1:N}$ are defined as ${}^{l}n_{1:N}≜[\begin{array}{ccc} {}^{l}n_{1} & \cdots & {}^{l}n_{N} \end{array}]$. Here, the second moment matrix (${}_{r}^{l}M\in R^{3\times 3}$) is constituted by

(1) $${}_{r}^{l}M=E\left[{}^{l}n_{1:N}{}^{l}n_{1:N}^{⊤}\right]=\frac{1}{N}{}^{l}n_{1:N}{}^{l}n_{1:N}^{⊤}.$$

To detect the rank and ratio of its eigenvalues, let us conduct the eigenvalue decomposition as

(2) $$\begin{array}{cc} {}_{r}^{l}M & =V\Lambda V^{⊤}\\ & =\left[\begin{array}{ccc} | & | & |\\ v_{1} & v_{2} & v_{3}\\ | & | & | \end{array}\right]\left[\begin{array}{ccc} \lambda_{1} & 0 & 0\\ 0 & \lambda_{2} & 0\\ 0 & 0 & \lambda_{3} \end{array}\right]V^{⊤}, \end{array}$$

where V is the orthonormal square matrix whose columns are eigenvectors $v_{i}$(i=1,2,3) and Λ is the diagonal matrix whose elements are associated eigenvalues ($\lambda_{i}$). For convenient representation, eigenvectors are sorted according to their eigenvalues in descending order ($\lambda_{1}\geq \lambda_{2}\geq \lambda_{3}$). These eigenvalues are equal to or greater than 0 because ${}_{r}^{l}M$ is a positive semi-definite matrix.

Now, let us exploit the ratio of eigenvalues ($\lambda_{2}/\lambda_{1}$). Its purpose is to discriminate the effective rank. Note that the real and false 2-pair correspondences can be detected by evaluating this ratio as its value tends to be close to 0 (i.e., $\lambda_{1}\gg \lambda_{2}$) for false cases but higher than a certain threshold for real cases.

Last but not the least, the remaining task is to select a proper threshold considering sensor noise level. For Kinect v2, it has been shown that its depth distortion yields a fluctuation error within ±6 mm [27]. With this amount of distortion, rotational covariance between poses is evaluated to be 4° for our plane-based method. In accordance with the empirical analysis, let us assume that there are two unit normals $\hat{n}={\left[\begin{array}{ccc} 1 & 0 & 0 \end{array}\right]}^{⊤}$ and ${\hat{n}}^{*}={\left[\begin{array}{ccc} \cos4{}^{\circ} & -\sin4{}^{\circ} & 0 \end{array}\right]}^{⊤}$ of which the included angle is 4°. Then, the value of the second moment matrix for $\hat{n}$ and ${\hat{n}}^{*}$ can be calculated as

(3) $$\begin{array}{ccc} M_{\hat{n}{\hat{n}}^{*}} & = & \frac{1}{2}[\begin{array}{cc} 1+\cos^{2}4{}^{\circ} & -\sin4{}^{\circ}\cos4{}^{\circ}\\ -\cos4{}^{\circ}\sin4{}^{\circ} & \sin^{2}4{}^{\circ} \end{array}]\\ & = & V\left[\begin{array}{cc} 9.988\times 10^{-1} & 0\\ 0 & 1.2\times 10^{-3} \end{array}\right]V^{⊤} \end{array}$$

Here, note that the dimensionality of the second moment matrix is reduced from 3 to 2 because the number of the sample ($\hat{n}$, ${\hat{n}}^{*}$) is smaller than the dimension of vector space ($R^{3\times 1}$). Thus, using these eigenvalues in Equation (3), we set the threshold of the ratio to be 1.2 × 10${}^{-3}$ (≒ 1.2 × 10${}^{-3}$/9.988 × 10${}^{-1}$).

### 2.2. Compensation Method for Degenerate Rotation

**Figure 3:**
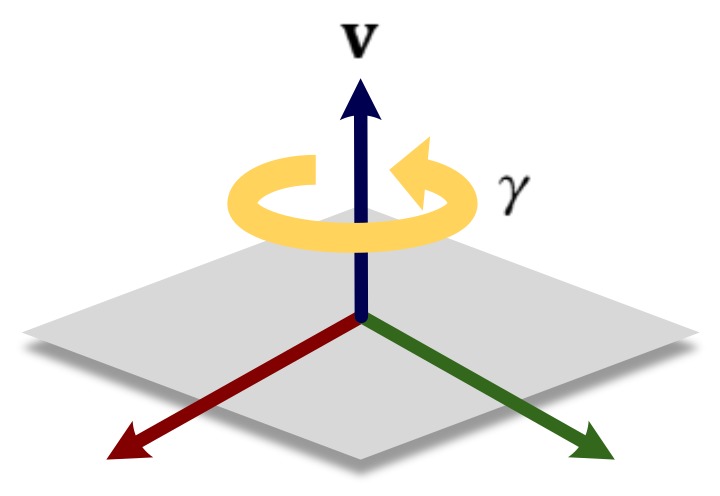
Illustration of rotational singularity under the constraint of 1-pair correspondence.

In the 1-pair correspondence case, as shown in Figure 3, the amount of rotation (γ) normal to the plane (v) cannot be estimated, where v corresponds to the one effective eigenvector of the second moment matrix in Equation (2). Here, IMU measurements are only used for compensating an ill-conditioned component regarding γ of the state that cannot be estimated by the features. For other well-conditioned components of which value can be estimated from the feature, measurements of IMU are not utilized due to their inaccuracy compared to those of features.

Before looking deep into mathematical details, let us show some quaternion definitions. A unit quaternion ($q\in R^{4}$) for the relative rotation is represented by

(4) $$q≜q_{0}+q_{x}i+q_{y}j+q_{z}k=\langle q_{0},\vec{q}\rangle ,$$

where $q_{0}$ and $\vec{q}$ are scalar and vector parts, respectively. The conjugate of q is

(5) $$q^{*}=\langle q_{0},-\vec{q}\rangle .$$

The product of two quaternions p and q is

(6) $$p⊗q≜\langle p_{0}q_{0}-\vec{p}\cdot \vec{q},p_{0}\vec{q}+q_{0}\vec{p}+\vec{p}\times \vec{q}\rangle ,$$

where · is the dot product and × is the cross product.

The main idea of this subsection is to set γ by projecting the IMU’s estimation in a way that it becomes parallel to the plane. In other words, this idea is a hybrid of two different estimations. One prediction is derived from the plane-based estimation. It keeps the plane constraint. However, it has an uncertain γ value. The other is derived from the IMU estimation. It does not necessarily keep the plane constraint. However, it has accurate γ value (at least for the given short time interval).

Now, let us denote the IMU prediction to be $q_{\mathrm{imu}}$ and the amount of rotation that projects $q_{\mathrm{imu}}$ parallel to the plane to be Δq. Here, Δq can be derived from two normal vectors, $v_{\mathrm{imu}}$ and $v_{\mathrm{reg}}$. They are updated normal vectors by IMU ($q_{\mathrm{imu}}$) and plane information ($q_{\mathrm{reg}}$), respectively:

(7) $$\langle 0,v_{\mathrm{imu}}\rangle =q_{\mathrm{imu}}⊗\langle 0,v\rangle ⊗q_{\mathrm{imu}}^{*},$$

(8) $$\langle 0,v_{\mathrm{reg}}\rangle =q_{\mathrm{reg}}⊗\langle 0,v\rangle ⊗q_{\mathrm{reg}}^{*}.$$

Now, Δq can be calculated by projecting $v_{\mathrm{imu}}$ parallel to $v_{\mathrm{reg}}$ in the shortest arc length as

(9) $$\Delta q=\frac{\langle v_{\mathrm{imu}}\cdot v_{\mathrm{reg}}+∥v_{\mathrm{imu}}∥∥v_{\mathrm{reg}}∥,v_{\mathrm{imu}}\times v_{\mathrm{reg}}\rangle }{∥\langle v_{\mathrm{imu}}\cdot v_{\mathrm{reg}}+∥v_{\mathrm{imu}}∥∥v_{\mathrm{reg}}∥,v_{\mathrm{imu}}\times v_{\mathrm{reg}}\rangle ∥}.$$

### 2.3. Further Processes

Although the orientation in the degeneracy direction is compensated for by Equation (9), a translation in that direction is still in degeneracy. Pathak et al. [17] have proposed a method that corrects uncertain translation by using IMU’s acceleration measurements. We also adopted this method for translation.

For loop-closure, we used a 3D Gestalt descriptor based method proposed in [25]. Finally, for back-end optimization, we implemented IRLS (iteratively reweighted least squares) that excluded less accurate outliers as in [26].

## 3. Experiments

**Figure 4:**
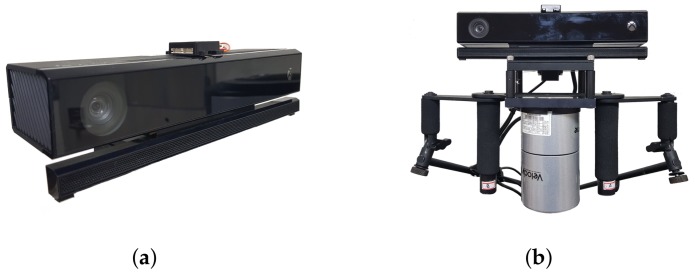
Two different sensor systems used in the experiment. (**a**) a hand-held low-cost sensor system; (**b**) a backpack high-cost sensor system. The high-cost system is rigidly equipped with the low-cost system for rigorous validation.

For performance validation, SLAM experiments were conducted with two different sensor systems as shown in Figure 4. One (Figure 4a) is a low-cost sensor system with a Kinect v2 and cheap IMU (CH-UM7: ±4° for dynamic pitch/roll accuracy, ±8° for dynamic yaw accuracy). The other (Figure 4b) is a high-cost sensor system [28] with Velodyne LiDAR (HDL-32E) and MicroStrain IMU (3DM-GX3-45: ±2° for dynamic pitch/roll/yaw accuracy). The purpose of the high-cost system is to extract the ground truth.

**Figure 5:**
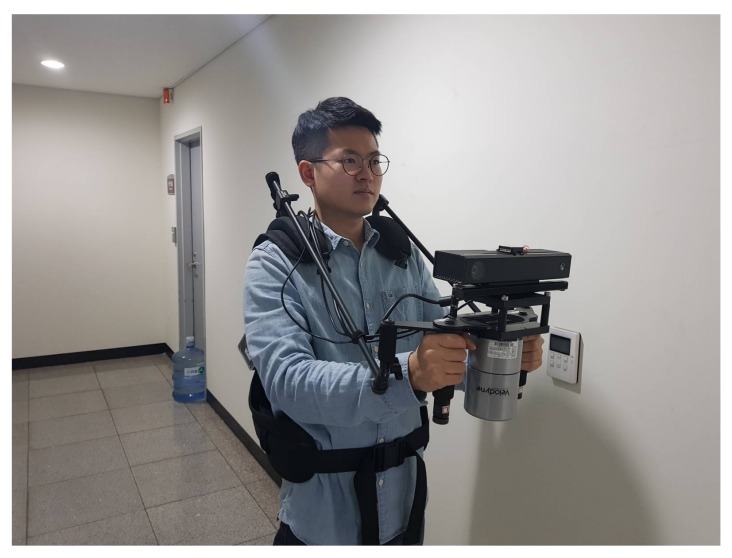
The sensor system used to acquire several datasets.

For data acquisition, an operator carried the system (as shown in Figure 5) through a small-sized building with narrow pathways. The operator navigated 62 m. A total of 410 place indices were generated. As a result, a graph was built where 255, 145, and nine edges were in 3-pair, 2-pair, and 1-pair plane correspondences, respectively.

Given the information, two methods were applied. One was a plane-IMU integrated (pl-IMU) method, which was an extension of the plane-based method [17] in a way that IMU information could be embedded. The other was our proposed method, which was identical to the previous method except that 1-pair corresponding degeneracy was detected and compensated for.

**Figure 6:**
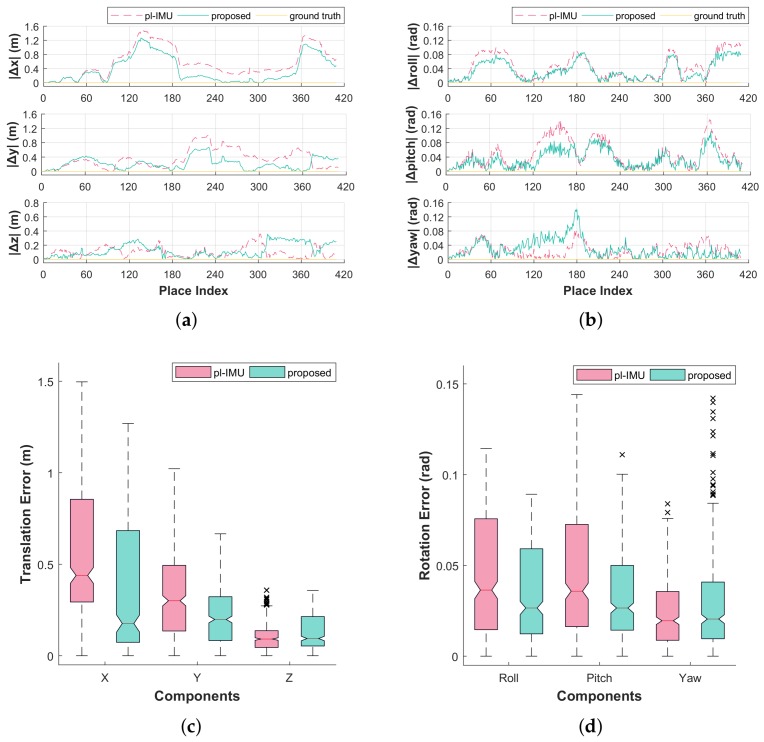
Graphs and box-plots for local pose error compared to the ground truth for each compensation method (pl-IMU and our proposed method). (**a**) local errors of translational components; (**b**) local errors of rotational components; (**c**) box-plot of translation error; (**d**) box-plot of rotation error.

**Figure 7:**
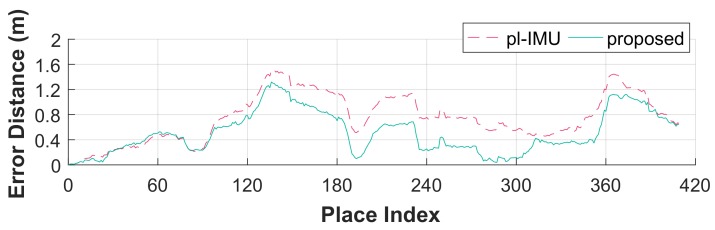
Local error distances compared to the ground truth for each compensation method.

As shown in Figure 6, the accuracy of all components was increased except for the two components *z* and *yaw* (of which median values are similar, respectively, in both methods). Figure 7 shows error distances of the two methods relative to the ground truth for 3D pose states. Error averages were significantly reduced using the proposed method as shown in Table 1.

Here, note that compensating rotation decreased both translation and rotation errors. This is a natural consequence as graph optimization increases the overall map accuracy due to rotational updates.

**Figure 8:**
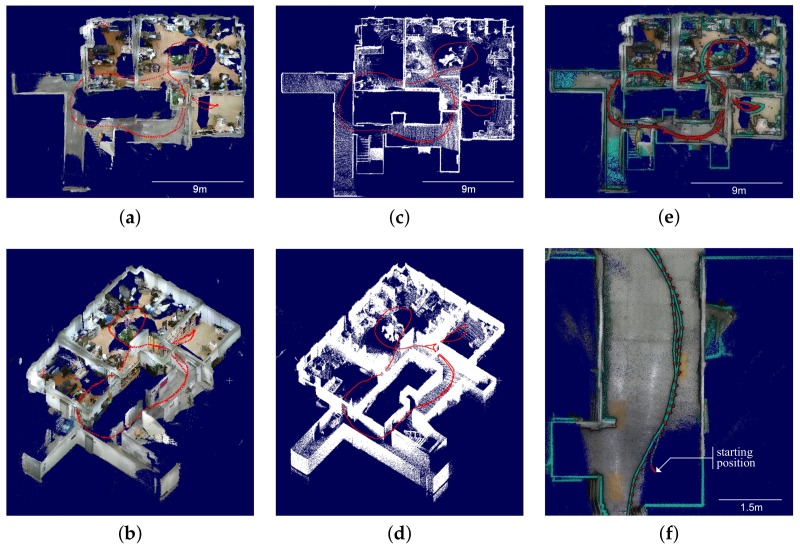
Trajectories and mapping results with truncated ceiling. (**a**) optimized map top view; (**b**) optimized map bird’s-eye view; (**c**) ground truth map top view; (**d**) ground truth map bird’s-eye view; (**e**) superimposed map top view; (**f**) superimposed map close-up view. (**a**,**b**) display RGB-D map optimized by the proposed method using low-cost sensor system. (**c**,**d**) show ground truth acquired using high-cost sensor system. (**e**,**f**) show superimposed map between the optimized map and the ground truth, where green-colored point cloud and trajectory are the ground truth. To improve depth visualization, an eye-dome lighting shader is applied to (**e**,**f**) where the point cloud of ground truth is randomly subsampled by 10%.

The performance can also be verified by 3D mapping. The map created by the proposed method showed high-quality consistency (Figure 8a,b), similar to that shown by the ground truth (Figure 8c,d). Figure 8e shows a top view where two trajectories, corresponding to the ground truth (green line) and the estimation of the proposed method (red dot), are superimposed. In Figure 8f, it can be verified that the loop closure is successfully conducted where accumulated errors in Figure 8e are significantly redeemed.

## 4. Conclusions

This paper proposed a degeneracy detection and compensation method for orientations that could arise when 3D SLAM algorithms used plane features. This degeneracy is induced when less than three plane-pairs are detected for two consecutive poses. It has significant correlation with the narrowness of a sensor’s field of view or the target environment. Our experiment showed that, when a 550 m^2^ indoor space was mapped with a Kinect v2, 37.7% of data acquisition poses were in a degeneracy situation.

The proposed method detected degeneracy by using the rank of the second moment matrix constituted by plane normals. To compensate orientations encountered by the degeneracy, IMU orientations were projected in a direction that was orthogonal to non-degenerate orientations.

## Figures and Tables

**Table 1:** Average errors of 3D poses for two different methods.

	Translation Error Avg. (m)			Rotation Error Avg. (rad)		
	X	Y	Z	Roll	Pitch	Yaw
pl-IMU	0.5773	0.3466	0.1004	0.0445	0.0468	0.0241
proposed	0.3714	0.2164	0.1266	0.0347	0.0338	0.0286
